# Comparison of Four Oil Extraction Methods for Sinami Fruit (*Oenocarpus mapora* H. Karst): Evaluating Quality, Polyphenol Content and Antioxidant Activity

**DOI:** 10.3390/foods11101518

**Published:** 2022-05-23

**Authors:** Ana María Muñoz, Sandra Casimiro-Gonzales, Raquel B. Gómez-Coca, Wenceslao Moreda, Ivan Best, María Isabel Cajo-Pinche, Juan Francisco Loja, Elena Ibañez, Alejandro Cifuentes, Fernando Ramos-Escudero

**Affiliations:** 1 Instituto de Ciencias de Los Alimentos y Nutrición, Universidad San Ignacio de Loyola (ICAN-USIL), Campus Pachacamac, Sección B, Parcela 1, Fundo La Carolina, Pachacamac, Lima 15823, Peru; amunoz@usil.edu.pe (A.M.M.); scasimiro@usil.edu.pe (S.C.-G.); ibest@usil.edu.pe (I.B.); 2 Unidad de Investigación en Nutrición, Salud, Alimentos Funcionales y Nutraceúticos, Universidad San Ignacio de Loyola (UNUSAN-USIL), Av. La Fontana 750, Lima 15024, Peru; 3 Instituto de la Grasa, CSIC, Campus Universidad Pablo de Olavide, Building 46, Ctra. de Utrera km 1, 41013 Sevilla, Spain; raquel.coca@ig.csic.es (R.B.G.-C.); wmoreda@ig.csic.es (W.M.); 4 Carrera Profesional de Ingeniería Agroindustrial, Universidad Nacional Amazónica de Madre de Dios (UNAMAD), Jr. Jorge Chávez 1160, Puerto Maldonado 17001, Peru; cajo_maisa@hotmail.com; 5 Asociación para la Conservación de la Cuenca Amazónica (ACCA), Madre de Dios 17001, Peru; jloja@conservacionamazonica.org; 6 Foodomics Laboratory, Instituto de Investigación en Ciencias de la Alimentación (CIAL, CSIC-UAM), Nicolás Cabrera 9, Campus de Cantoblanco, 28049 Madrid, Spain; elena.ibanez@csic.es (E.I.); a.cifuentes@csic.es (A.C.); 7 Facultad de Ciencias de la Salud, Universidad San Ignacio de Loyola, Av. La Fontana 750, Lima 15024, Peru

**Keywords:** sinami oil, oil extraction, physicochemical composition, carotenoids, chlorophylls, color measurement, chemometrics

## Abstract

The sinami palm (*Oenocarpus mapora* H. Karst) is a plant from the South American Amazonia that has great potential for industrial applications in the development of functional foods, nutraceuticals and cosmeceuticals. In this manuscript, the physicochemical properties, total polyphenol content and antioxidant activity of sinami oil that was obtained using four extraction systems, namely expeller press extraction (EPE), cold press extraction (CPE), ultrasound-assisted extraction (UAE) and supercritical fluid extraction (SFE), were studied and compared. The oxidative stability (OSI) was statistically non-significant in EPE and SFE. The chromatic properties (CIELab) were influenced by the extraction methods and SFE presented high values of L* and a lower content of plant pigments. Ultrasound-assisted extraction showed a higher content of polyphenols and higher antioxidant activity. Different analyses for the evaluation of the physicochemical properties, the content of total polyphenols and antioxidant activity were used to classify sinami oil according to chemometrics using principal component analysis (PCA). For example, the sinami oil that was obtained using each extraction method was in a different part of the plot. In summary, sinami oil is an excellent resource for plant pigments. Additionally, the information that was obtained on the quality parameters in this study provided a good foundation for further studies on the characterization of major and minor compounds.

## 1. Introduction

A variety of palm species grow in the South American Amazonia, including aguaje or buriti (*Mauritia flexuosa*), ungurahui (*Oenocarpus bataua* C. Martius), pijuayo or pupunha (*Bactris gasipaes*), huasai or açai (*Euterpe precatoria*, *Euterpe oleracea*), macaúba (*Acrocomia aculeata*), bacaba-de-leque (*O. distichus*), babassu (*Attalea speciosa*), bacaba (*O. bacaba*), buritirana (*Mauritiella armata* Mart.), babassu (*Orbignya phalerata* Mart.), juçara (*Euterpe edulis* Martius) and sinami (*O. mapora*). These palms are a source of oils that can be used for human consumption and in the food and cosmetics industries [1,2,3,4,5,6,7,8].

Little is known about sinami fruit. Phenological studies have been recently conducted in the Madre de Dios region of Peru to determine the flowering and fruiting periods of *O. mapora* H. Karst [7]. In the South American Amazonia and the Peruvian rainforest, sinami fruit is consumed as a fresh fruit or as chonta palm and is used to prepare soft drinks and edible extract oil [9]. The general chemical composition of its pericarp includes water (63.31%), ash (0.53%), lipids (12.34%), proteins (2.74%), fibers (14.83%) and carbohydrates (6.26%). Other components include 972 mg of gallic acid equivalents (GAE),100 g (dry weight) of total polyphenols, 49.91 mg to 100 g (dry weight) of total anthocyanins and 17.36 and 53.04 mg/g (dry weight) of antioxidant capacity, which is expressed as Trolox equivalent using the DPPH and ABTS methods, respectively [10]. The chemical characterization and evaluation of the antioxidant capacity of different *Oenocarpus* species (*O. bataua*, *O. distichus* Mart. and *O. bacaba*) have been reported. These plants are rich in phenolic compounds, especially anthocyanins, condensed tannins, stilbenes, flavanols and phenolic acids [11,12,13,14].

Edible oils can become rancid and lose their nutritional value and sensory qualities. Oil quality can be influenced by the harvesting and post-harvesting processes of the fruit, as well as the extraction, handling and storage processes of the oil. Other factors that are also associated with rancidity and deterioration over time are moisture content, microbial load, light exposure and the presence of metals that can enhance catalytic oxidation in oils and chemicals, such as pesticides and mycotoxins [15,16]. The assessment and frequent monitoring of the quality parameters of edible oils as criteria for quality control are essential to ensure food safety and promote consumer health. Several analytical methods have been proposed to evaluate the quality parameters of edible oils. The physicochemical properties that are assessed include free fatty acids, peroxide value, conjugated dienes and trienes, moisture, saponification, iodine content, color, specific gravity, smoke point, p-anisidine content, carbonyl content and polar compounds, among others [17,18,19]. These analytical measurements provide information about unsaturation, the initial or primary formation of oxidation products from unsaturated fatty acids and free fatty acid formation due to oxidative rancidity or oxidation. The physical and chemical properties of edible oils and fats can affect the quality of oil-based foods and should be thoroughly analyzed [18]. Moreover, oxidative stability has been considered one of the main quality parameters of edible oils [20]. Oxidative stability is usually measured using the Rancimat method, which is conducted over short time periods and at high temperatures. The Rancimat stability of virgin oils, such as olive oils with different harvesting times, ranges from ~25 to 150 h [21]. The oxidative stability of virgin oils depends on their components, especially tocopherols, polyphenols and pigments, such as plant chlorophyll and carotenoids. A direct correlation between Rancimat stability and total polyphenol content has been reported [21], which indicates that resistance to oxidation could be due to the higher antioxidant contents of virgin oils. Several natural antioxidants that are found in edible oils, such as tyrosol, hydroxytyrosol, rutin, quercetin and oleuropein and vanillic, caffeic, p-coumaric, ferulic and sinapic acids, have proven to be excellent molecules that increase induction time and improve quality indicators [21,22,23,24,25].

The different methods that are used for the extraction of edible oils include mechanical extraction (expeller pressing and cold pressing) and green extraction methods (ultrasound-assisted, microwave-assisted, supercritical fluid, subcritical and enzyme-assisted aqueous extraction) [26,27,28,29,30], which have attracted considerable attention due to their environmental friendliness. Oils that are obtained using these techniques are richer in bioactive substances, namely plant pigments, phenolic compounds, phytosterols, tocopherols, tocotrienols and aromatic compounds, which contribute to the sensory qualities of the oil [31,32,33] and are considered to be health promoters [16].

This study analyzed the extraction of oil from sinami fruit (*O. mapor*a H. Karst) using four different extraction methods: expeller press extraction (EPE), cold press extraction (CPE), ultrasound-assisted extraction (UAE) and supercritical fluid extraction (SFE). Then, the oils were compared in terms of their physicochemical properties, total polyphenol content and antioxidant activity. All of the studied variables were subjected to principal component analysis (PCA) in order to identify any similarities and differences between the oils.

## 2. Materials and Methods

### 2.1. Sampling and Sample Treatment

**Figure 1:**
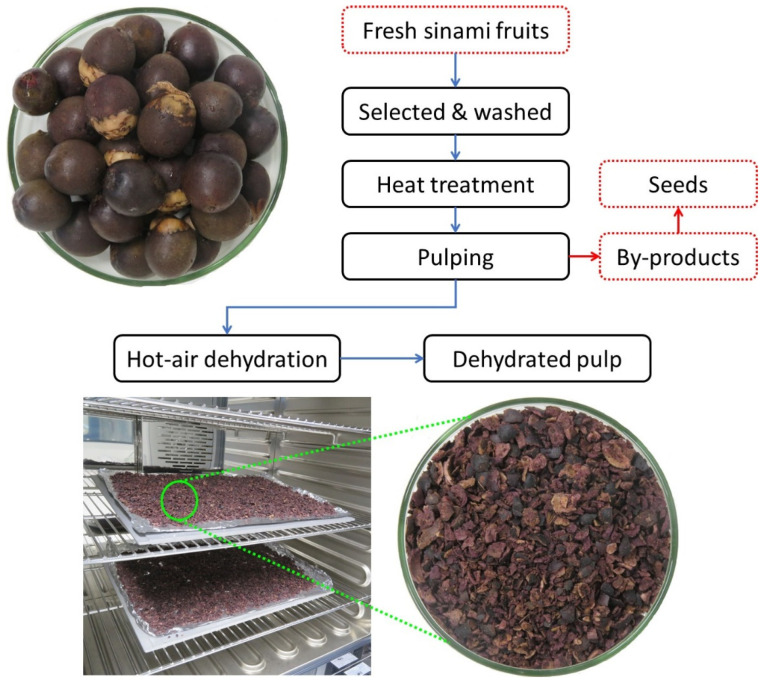
Flowchart of the process for obtaining dehydrated pulp from sinami fruit.

Fresh sinami fruit was harvested and supplied by Haktone Wabakae Kate’dapi Uruanda/Wara (HAWAKAUR E.I.R.L; Tambopata, Madre de Dios, Peru) in January 2021. The fruit was selected, washed and heat-treated at 100 °C for 15 min in order to easily obtain the pulp. Pulping was performed manually using a stainless steel knife and the fresh pulp was then dehydrated at 100 °C for 2 h in a forced air oven (UF160, Memmert, Schwabach, Germany) (Figure 1). The samples were vacuum-packed (SU-316, Sammic, IL, USA) and stored at −20 °C (A54546, Infraca, Valencia, Spain) until oil extraction.

### 2.2. EPE Method

Sinami oil was mechanically extracted from the dehydrated pulp using expeller pressing (oil press machine, Lima, Peru). Briefly, 500 g of dehydrated pulp was fed into the expeller press at 90 °C and crushed. The crushed material was placed in an Erlenmeyer flask and agitated in a water bath at 30 °C. Water was added at a ratio of 1:3 (sample:water) and incubated at 60 °C under agitation for 24 h. The samples were transferred to 50-mL polypropylene centrifuge tubes and centrifuged at 5000 rpm and 25 °C for 30 min (5810R, Eppendorf, Hamburg, Germany). The oil that was extracted was placed in amber glass containers and stored at −20 °C (A54546, Infraca, Valencia, Spain) until further use.

The Oil yield (%) was calculated based on the ratio between the mass of the oil that was obtained ($m_{oil}$) and the initial mass of the dehydrated pulp ($m_{initial\;mass}$) using the following equation:

(1) $$Oil\;yield\;\left(\%\right)=\frac{m_{oil\;\left(g\right)}}{m_{initial\;mass\;\left(g\right)}}\times 100$$

### 2.3. CPE Method

Approximately 400 g of dehydrated sinami pulp was placed inside a filter bag in the extraction cylinder of a hydraulic press at 35.5 °C and 4.1 MPa. The oil that was obtained was stored at −20 °C until further use.

### 2.4. UAE Method

An ultrasound bath at 40 KHz that had an electrical output power of 160 W (CPX5800H-E, Bransonic, Danbury, CT, USA) was used. Briefly, ethanol was added to 25 g of dehydrated sinami pulp (sample:ethanol ratio of 1:12) in an Erlenmeyer flask, which was then placed in a water tank. Extraction occurred at 40 °C for 60 min. The solvent was then removed using a rotary evaporator (R-300, Buchi, Flavil, Switzerland) at 100 mbar, 60 °C and 50 rpm. The oil that was obtained was stored at −20 °C until further use.

### 2.5. SFE Method

Sinami oil was extracted with supercritical carbon dioxide (sc-CO_2_) using a supercritical fluid extractor Spe-ed ^TM^ (Helix, Applied Separations, Allentown, PA, USA) that was equipped with a CO_2_ cylinder (99.5% purity, Ryso SAC., Lima, Peru), a 175 L air compressor with a maximum allowable working pressure of 10.62 bar (model PRPD180MP-019, Maringá, PR, Brazil), a recirculating chiller (240 V; 50 Hz AC voltage) with a cooling solution of 50% propylene glycol USP and 50% water at 4 °C (model 7027, PolyScience, Niles, IL, USA) and a valve for the mass flow control of carbon dioxide at the system outlet (Alicat Scientific, Arizona, AZ, USA). The extraction vessel capacity was 1000 mL, which included a 1000-mL internal basket. Briefly, approximately 307 g of ground dehydrated pulp was loaded into the inner basket, which was then placed inside the extraction vessel. The extraction conditions were 60 °C, 35 MPa and 100 °C at the micro metering valve. The extraction was performed over static and dynamic periods, as proposed by Cunha et al. [34], except the depressurization period was modified to 60 min (Figure S1). The oil that was obtained was stored at −20 °C until further use.

### 2.6. Quality Assessment

The relative density of the oils that were obtained using the four extraction methods was estimated at 20 °C, as described by Zahir et al. [35]. The refractive index was determined at 20 °C using an analog ABBE refractometer (NAR-1T LIQUID, Atago Co., Tokyo, Japan). The conjugated dienes and trienes were determined according to the IOC [36]. Briefly, approximately 0.1 g of oil was placed in a volumetric flask and diluted with cyclohexane. The absorbance of the resulting clear solution was measured at 232, 266, 270 and 274 nm using a Jasco spectrophotometer (V-770, Jasco, Tokyo, Japan). The extinction coefficient values (K232 and K270) and the extinction coefficient variation (∆*K*) were calculated using Equations (2) and (3). To determine the free acidity (FFA), 10 mL of ethanol–diethyl ether (1:1 *v*/*v*) was added to 1.0 g of oil and titrated with 0.088 M of potassium hydroxide. The titration of the free fatty acids was performed using a pH meter (pH 7110, InoLab, Wellheim, Germany) until the color of the phenolphthalein changed at pH 8.2. The results were expressed as a percentage of oleic acid (the main fatty acid in sinami oil). The peroxide value (PV) was determined as described by OJEU [17]. Briefly, a solution of acetic acid–chloroform and a saturated solution of potassium iodide were added to 1.0 g of oil and titration was conducted using 0.01 N of sodium thiosulfate. The results were expressed as mEq of active oxygen/kg fat. The AOCS [37] official method Cd 18–90 was used to determine the p-anisidine value (pAV). The TOTOX value was calculated using Equation (4) [38]. The saponification value (SV) was determined according to the AOCS [37] official method Cd 3–25.

(2) $$K_{232\;or\;270}=\frac{A_{232\;or\;270}}{C_{\;x}\;L}$$

(3) $$\Delta K=K_{270}-\frac{\left(K_{266}+K_{274}\right)}{2}$$

(4) TOTOX=2×PV+pAV

where $A_{232}$ and $A_{270}$ are the absorbance values at the respective wavelengths (232 and 270 nm), C is the sample concentration (g/100 mL) and L is the optical path length (cm).

### 2.7. Oxidative Stability Using the Rancimat Method

The oxidative stability of the oils was determined using a Metrohm Rancimat analyzer (892 Professional Rancimat, Metrohm Instruments, Herisau, Switzerland). Reaction vessels that contained approximately 2.5 g of oil were placed in a heating block at 110 °C and a temperature correction factor of 1.6 °C was used for each sample. Purified air was then bubbled at 20 L/h and a measuring vessel that contained 80 mL of deionized water was used to collect the short-chain volatile organic acids that were present in the effluent air [39,40]. Water conductivity and induction time (h) were automatically recorded using StabNet software, version 1.1.

### 2.8. Color Measurement

Color measurements were performed as previously described by Ramos-Escudero et al. [41], but with minor modifications. Briefly, aliquots of 300 μL of previously centrifuged (mini spin, Eppendorf, Hamburg, Germany) oil samples were placed in sample holders. For each sample, 10 photographs were taken under an OSRAM LED lamp (17 W, color temperature: 6500 K) using a digital camera (Canon, Power Shot SX60 HS, Tokyo, Japan). RGB-based color histograms were plotted using ImageJ 1.51j software (National Institutes of Health by Wayne Rasband, Bethesda, MD, USA). The RGB values were obtained from 15 multiple point selections per photograph. The RGB values were then converted into lab values using a color converter (Nix Color Sensor, https://www.nixsensor.com/free-color-converter/ (accessed on 7 February 2022)). For the input and output adjustments, the standard illuminant D65 and reference angle (daylight source) and 10° (angle of perception of a human observer) were taken into account. The chroma (C*ab) and hue (*h*ab) were determined using the equations described by Ramos-Escudero et al. [40].

### 2.9. Determination of Plant Pigment Content

Plant pigment content was determined using direct spectrophotometric measurement of the oil samples (Jasco spectrophotometer, V-770, Jasco, Tokyo, Japan). Spectra were recorded between 400 to 750 nm using a glass cuvette with a 2-mm optical path length. The carotenoid and chlorophyll contents were derived from Equations (5) and (6):

(5) $$Carotenoids\;\left(\mathrm{mg}/\mathrm{kg}\right)=\frac{A_{470}\times 10^{6}\;}{2000\times 100\times 0.2}$$

(6) $$Chlorophylls\;\left(\mathrm{mg}/\mathrm{kg}\right)=\frac{A_{670}\times 10^{6}\;}{613\times 100\times 0.2}$$

### 2.10. Determination of Total Polyphenol Content

Total polyphenol content was determined using the Folin–Ciocalteu assay. Briefly, 450 μL of toluene was added to 50 mg of oil. Then, 750 μL of the 0.2 N Folin–Ciocalteu reagent was added to 100 μL of each sample mixture and incubated for 5 min, followed by the addition of 750 μL of 7.5% sodium carbonate. After further incubation for 2 h in the dark, absorbance was measured at 725 nm. The total polyphenol content was estimated using a standard gallic acid curve (y = 0.0065x + 0.0367; R^2^ = 0.9996). The results were expressed as milligrams of gallic acid equivalents per kg of oil (mg GAE/kg).

### 2.11. DPPH Radical Scavenging Activity

The evaluation of antioxidant activity from DPPH was conducted according to Brand-Williams et al. [42]. Sample aliquots of 50 μL (working solutions: 100, 75, 50 and 25 mg of oil in 500 μL of toluene) were incubated with 950 μL of 100 μmol/L DPPH (in 60% ethanol) and vortexed constantly for 30 min (Thermo Scientific, Waltham, MA, USA). Absorbance was measured at 515 nm (Jasco spectrophotometer, V-770, Jasco, Tokyo, Japan). The results were expressed as inhibitory concentration 50% (IC_50-DPPH_).

### 2.12. ABTS Radical Scavenging Activity

The evaluation of antioxidant activity from ABTS radical scavenging was performed according to Miller and Rice-Evans [43]. Sample aliquots of 100 μL (working solutions: 100, 75, 50 and 25 mg of oil in 500 μL of toluene) were incubated with 900 μL of ABTS radical for 28 min with constant shaking, followed by centrifugation at 10,000 rpm for 2 min (mini spin, Eppendorf, Hamburg, Germany). Absorbance was measured at 734 nm. The results were expressed as inhibitory concentration 50% (IC_50-ABTS_).

### 2.13. Statistical Analyses

The results were shown as mean ± standard deviation (*n* = 3). A comparison of the means of the different variables was performed using an analysis of variance, followed by Duncan’s multiple range test when the significance level was *p* < 0.05. The contribution of the variables to the extraction methods was assessed using a PCA. Statistical analyses were performed using STATISTICA software, version 8.0 (StatSoft, Inc., Tulsa, OK, USA).

## 3. Results and Discussion

### 3.1. Quality Assessment of Oil

Table 1 summarizes the influence of the four extraction methods (EPE, CPE, UAE and SFE) on the physicochemical properties of the sinami oil that was obtained. In terms of yield, UAE showed the highest extraction capacity (15.64%), followed by SFE, EPE and CPE (7.45%, 6.99% and 5.52%, respectively). UAE has been used for the extraction of oils from fruit pulp [44], seeds [45] and agro-industrial by-products [46]. Yields ranging from 8 to 83% have been reported [47], with the highest values being obtained using ultrasonic cavitation. This phenomenon facilitates the release of the oil fraction from the plant cell wall, thereby allowing the extraction to be conducted at low frequencies [47,48].

Density plays an important role because it affects oil absorption and mass transfer rates during the cooling of frying oils [49]. The density of the sinami oil that was obtained using the different extraction methods ranged between 0.89 and 0.94 g/mL, with CPE and SFE showing the highest values. These density values were similar to those found in some other tropical oils, such as babassu, Brazil nut, buriti (or aguaje) and macadamia oils [50]. The refractive index is a physical property that is used in the analysis of fats and oils, which can provide information on the oil adulteration and purity, degree of unsaturation and fatty acid chain length [51]. The refractive index values of the sinami oil samples were between 1.4650 and 1.4691 and no significant differences were observed between the different extraction methods (*p* < 0.05). This was in agreement with previous reports of refractive index values of 1.47 in oils and fats from Amazonian plants, such as buriti oil (*M. flexuosa*) and patawa oil (*O. bataua*) [52]. The values of the spectrophotometric measurements K232, K270 and ∆K denote the quality characteristics and maturity index of fruit [53]. High values of these indicators in oils are related to their oxidation. The samples in this study showed K232 values between 3.10 and 3.93, K270 values between 0.35 and 0.77 and ∆K values between 0.006 and 0.018. The sinami oil that was obtained using CPE presented the lowest values, whereas the oil that was extracted using UAE showed the highest values. Moreover, ∆K values (from 232 and 270 nm) have been associated with variations in the formation of peroxide radicals and hydroperoxides during the oxidation of polyunsaturated fatty acids [54]. Increased K232 values have been related to inappropriate fruit storage, as well as unsuitable extraction conditions [55], whereas increased K270 values have been observed in stored oils or oils that were obtained from plant materials from previous harvests. Free fatty acids can act as pro-oxidants in oils due to their carboxyl groups, which is an indicator of oil deterioration. The sinami oil samples in this study contained between 0.55 and 1.05% free fatty acids, with the oils from CPE and EPE showing the lowest values and those from UAE and SFE presenting the highest vales. The observed differences in the free acid values could be associated with the effects of temperature. In addition, oils with high acidity show a lower resistance to oxidation than oils with low acidity [54]. The peroxide value (PV) is an indicator of initial oxidation. In addition, oils that were extracted at a high temperature have shown high PV values, triacylglycerol oxidation products and increased free fatty acid contents [29,56]. The PV values in our sinami oil samples were between 22.66 and 43.58 meq O_2_/kg. The sample that was obtained using UAE showed a high PV value, whereas the values were low for those that were obtained using CPE, EPE and SFE. According to Serra et al. [52], oils with PV values that are greater than 30 meq O_2_/kg exhibit poor oxidative stability, which can indicate that they have been in storage for long time or exposed to light. The p-anisidine value (pAV) is a measure of the aldehyde levels in oils that result from the reaction of p-anisidine with unsaturated carbonyl compounds. The TOTOX value is a measure of the overall oxidation state of the oil (a combination of PV and pAV) [54,57]. Our sinami oil samples showed pAV values between 2.11 and 4.13 and TOTOX values from 53.58 to 95.08. When only the p-anisidine assay was considered, EPE and CPE had the highest values. However, when the TOTOX value was taken into account, only the UAE sample showed a value of 95.08. The saponification value (SV) is a measure of the content of ester bonds and, therefore, is related to the fatty acids that are present in oils or fats (the fatty acids can be either free or esterified) [58]. The SV values of the different samples in this study ranged from 196.08 to 219.75 mg KOH/g. The lowest and highest values were observed in the UAE and SFE samples, respectively; the CPE and EPE samples showed no statistical differences. The SV values that have been reported for other edible oils from Amazonian palms were 183.91 and 181.52 mg KOH/g for buriti oil and patawa oil, respectively. Oleic acid (C18:1) is the main fatty acid in these oils (~78%) [52]. Cheikhyoussef et al. [59] reported saponification values between 172 and 199 mg KOH/g in oils.

### 3.2. Oxidative Stability of Sinami Oil

The oxidative stability index (OSI) is defined as the resistance of fats or oils to oxidation. Table 2 presents the oxidative stability of the sinami oil samples in this study. The oils that were obtained using EPE (5.53 h at 110 °C) and SFE (5.01 h at 110 °C) showed greater resistance to degradation from air oxidation than those that were obtained using CPE (2.69 h at 110 °C) and UAE (1.99 h at 110 °C). OSI values from 2.79 to 16.00 h have been reported for edible oils that were extracted from other tropical palms, such as seje (*Jessenia bataua*), macaúba (*Acrocomia aculeata*), bacaba (*O. bacaba*) and patawa (*O. bataua*) [2,4,60,61].

Ultrasound pre-treatment with different exposure times can affect the oxidative stability of olive oil; however, the quality characteristics and antioxidant activity are not affected by the exposure times [63]. Halim and Thoo [64] reported that the ultrasound treatment of palm oil and sunflower oil accelerates the lipid oxidation processes that have a significant effect on the generation of primary and secondary metabolites. Belayneh et al. [65] found that oxidative stability depends on the oil extraction method that is used and the composition of polar fractions, namely phenolic compounds and phospholipids. Increased antioxidant activity has been associated with the presence of polyphenols in oils, whereas an increase in the content of polyunsaturated fatty acids has been related to a decrease in the induction period.

### 3.3. Color Properties of Oil

The color of oils is an important parameter for quality control and is essential as a first criterion of evaluation [40,66]. The color measurements of the sinami oil samples in this study are shown in Table 3, where L* denotes oil brightness. Statistical differences were observed (*p* < 0.05). Based on this parameter, the samples could be arranged from lightest to darkest as SFE > CPE > UAE > EPE. CPE (63.28 ± 3.67 units) and UAE (63.20 ± 1.71 units) were statistically equal. In SFE, the L* value was high (82.70 ± 0.63 units) compared to the other extraction methods, which was probably associated with the lower content of plant pigments (Table 4). Low pigment extraction yields have been reported for SFE compared to UAE [67], which could account for the high clarity that was observed in the SFE samples.

**Figure 2:**
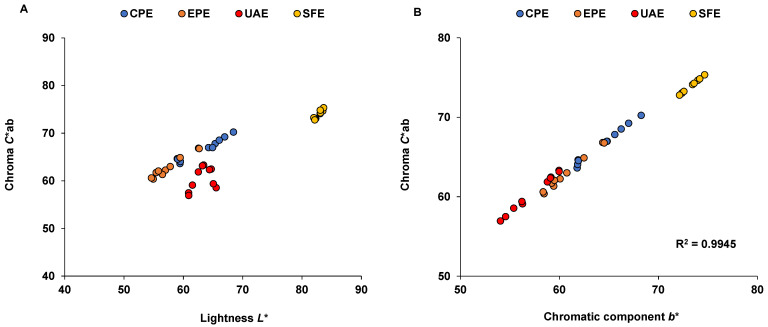
(**A**) Chroma vs. lightness plot and changes in the chroma values for the different sinami oil extraction systems; (**B**) correlation between chromatic parameter b* and chroma value in the sinami oil that was obtained using CPE, EPE, UAE and SFE.

The values that were obtained for the sinami oil that was extracted using SFE were similar to those that have been reported for olive oil (83.61–91.07 units) [68], whereas the values in the other samples were lower and, therefore, the oils were darker. In terms of chroma (C*ab), the mean values were between 60.48 and 73.05. The sinami oil that was extracted using SFE had the highest chroma values (72.80–75.36 units), i.e., it showed a higher degree of color deviation toward greater clarity (Figure 2A). The variation in chroma values between SFE and the nearest extraction method was ~6 units (CPE) and the difference from the farthest method was ~13 units (UAE). In addition, the parameter C*ab and the colorimetric coordinate b* showed higher variations for the samples from UAE (3.14) > EPE (2.29) > CPE (2.27), whereas the variation was 0.69 for the SFE sample. The variation was found to be greater in samples with higher plant pigment contents. A strong correlation was observed between the chroma value and the colorimetric coordinate b* (R^2^ = 0.9945) (Figure 2B). However, a negative correlation between C*ab and b* (r = −0.398) has been reported for the fruit of Chinese flame trees from different color groups [69] and it should be noted that anthocyanins were the main pigments in those samples. Increased chroma values and decreased b* values have been found in red-skinned fruit, whereas the increase is proportional between the C*ab and b* values in pink- and green-skinned fruit. Moreover, a high correlation between b* values and plant pigments has been observed in aguaje fruit, which is rich in carotenoids [70]. Likewise, correlation values of around 0.718 between plant pigments, chroma values and b* colorimetric coordinates have been reported in single varietal olive oils [68].

**Figure 3:**
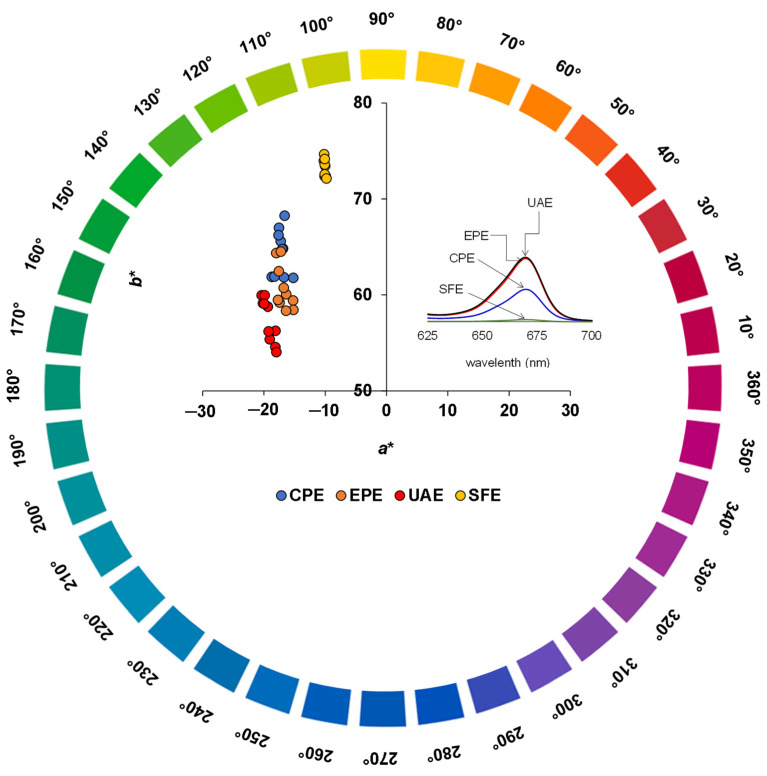
Colorimetric coordinates for the different sinami oil extraction systems within the a*b* plane.

The colorimetric coordinate a* ranged between −19.23 and −10.06 units for the different extraction methods, with UAE (−19.23) > CPE (−17.20) > EPE (−16.80). Values ranging from −3.84 to −0.24 units have been reported for some single varietal virgin olive oils [68], whereas other commercial oils, such as sacha inchi oil, have shown values between −62 and −3.62 units [40]. Kılıç et al. [71] reported a* values of −1.8 and −1.2 for corn oil and sunflower oil, respectively. The more negative a* values may be influenced by plant pigments, especially chlorophylls. Olive oils of different Italian varieties have shown a* values from −9.00 to −3.33 units [72]; therefore, negative values of a* correspond to a green color. In addition, a negative correlation between the chlorophyll content and a* value was found (r = −0.64). Nevertheless, it was also highlighted that the higher the chlorophyll content, the darker and greener the oils. The distribution of the sinami oils that were extracted using the different methods could be observed in the a*b* plane. The oils were found in the second quadrant, which corresponded to negative values of a* and positive values of b* (Figure 3). In terms of *h*ab, the values were between 71.46° and 82.17°, thus placing the sinami oils that were extracted using UAE, CPE, EPE and SFE in the green-yellow color area. The yellowest sinami oil was obtained using SFE, whereas the oils that were derived using CPE (75.03°) and EPE (74.54°) were statistically equal. Olive oils have shown *h*ab values of between 90.77° and 95.95° [68].

### 3.4. Plant Pigments, Total Polyphenols and Antioxidant Activity of Sinami Oil

As shown in Table 4, the oils that were obtained using EPE and UAE had the highest total plant pigment levels, followed by those that were obtained using CPE and SFE (161.18, 153.99, 103.57 and 15.78 mg/kg, respectively). The EPE, UAE and CPE samples presented chlorophyll contents that were higher than their total carotenoid contents. Conversely, the carotenoid content in the SFE sample was approximately four times higher than the total chlorophyll content. Low yields of plant pigments have been previously described when using supercritical carbon dioxide extraction. For example, yields of around 1% were reported for *Bixa orellana* seeds after extraction at 200 bar and 40 °C [73]. Kultys and Kurek [74] argued that the extraction of carotenoids using SFE produces low yields due to the high molecular weight of the extractable compounds. Nevertheless, the use of polar solvents, such as ethanol, increases the recovery of plant pigments. For example, approximately 97.1% of carotenoids (astaxanthin, lutein and β-carotene) can be extracted through SFE by using ethanol as a co-solvent [75]. Our results were in agreement with previous reports of high yields when using EPE, CPE and UAE [76,77].

The phenolic compounds in oils exhibit different properties, including antioxidant activity, anti-inflammatory activity, neuroprotective activity and immunomodulatory activity. The total polyphenol contents and antioxidant activity of the sinami oil samples in this study are shown in Table 4. The total polyphenol contents ranged from 89.03 to 615.18 mg/kg, with UAE > SFE > EPE > CPE. As expected, the extraction yield from UAE when using ethanol as a solvent was around seven times higher than that from CPE, around five times higher than that from EPE and around three times higher than that from SFE. With regard to the antioxidant activity, which was evaluated using the DPPH and ABTS methods, the IC_50_ values were between 3.24 and 5.70 mg/mL and 7.81 and 13.12 mg/mL, respectively. The ratio of DPPH antioxidant activity to polyphenol content was 0.6135, whereas the ratio for ABTS to total polyphenols was 0.9891. The ratio of DPPH to ABTS was 0.7115. The antioxidant activity that was measured using DPPH (IC_50_) for the sinami oils that were obtained using the different extraction methods was higher than that for other oils, such as sunflower (26.9 mg/mL), sesame (11.7 mg/mL), virgin olive (15.5 mg/mL), soybean (17.2 mg/mL) and corn (34.7 mg/mL) oils [78]. The higher content of polyphenols in the UAE sample when using ethanol was explained by the polarity of the solvent and the higher affinity for phenolic compounds [79]. Furthermore, cavitation allowed for increased mass transfer, the penetration of the solvent into the oily matrix and the release of polyphenols into the polar fraction. The polyphenol content of several tropical palm oils has been determined, such as the different morphotypes of aguaje oils (153–328 mg GAE/kg) [41], virgin babassu oil (~1100 mg GAE/kg) [5] and seje oil (31–64 mg caffeic acid/kg) [2]. Our results for the polyphenol contents of the sinami oils that were obtained using the different extraction methods showed significant differences (*p* < 0.05). Therefore, the extraction method affected the total polyphenol content of the extracted oil.

### 3.5. PCA Analysis

**Figure 4:**
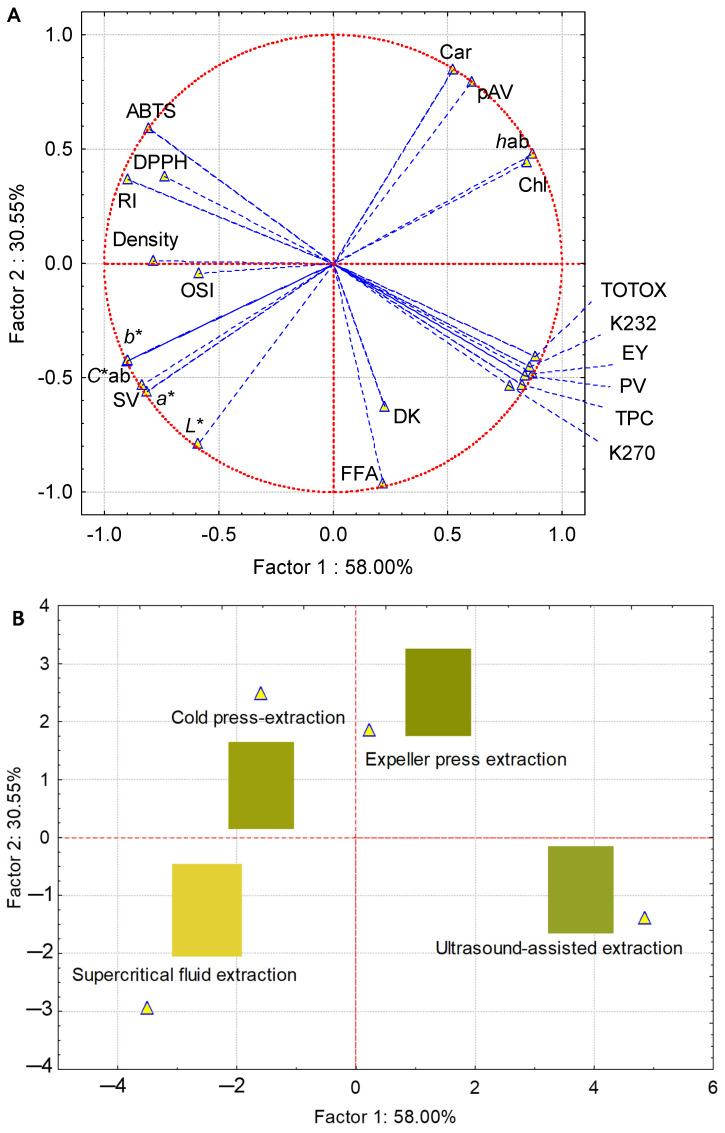
PCA of the sinami oils that were obtained using CPE, EPE, UAE and SFE, based on the physicochemical properties, total polyphenol content and antioxidant activity: (**A**) PCA score plot with PC1 and PC2, based on the dataset of 22 different variables of plant pigments (Car, carotenoids; Chl, chlorophylls), chromatic parameters (L*; a*; b*; C*ab; *h*ab), physicochemical properties (EY, extraction yield; density; RI, refractive index; K232 and K270, dienes and trienes; ∆K, extinction coefficient variation; FFA, free fatty acidity; PV, peroxide value; pAV, p-anisidine value; TOTOX, total oxidation value; SV, saponification value; OSI, oxidative stability index) and antioxidant activity (DPPH; ABTS; TPC, total polyphenol content); (**B**) PCA classification for the same sinami oils.

For the principal component analysis (PCA), the following variables were used: chromatic parameters (L*, a*, b*, C*ab and *h*ab), quality parameters (extraction yield, density, refractive index, specific extinction (K232), specific extinction (K270), extinction coefficient variation (∆K), free acidity, peroxide value, p-anisidine value, TOTOX and saponification value), plant pigments (carotenoids and chlorophylls), total polyphenol content and antioxidant activity (DPPH and ABTS). As shown in Figure 4A, the first two principal components explained 88.55% of the total variance. PC1 represented 58.00% of the total variance and showed a positive correlation with the refractive index (RI) and antioxidant activity using ABTS. PC2 described 30.55% of the total variance and showed a positive correlation with the chromatic variables (L*, a*, b* and C*ab) and the saponification value (SV), while K232, PV, TOTOX, TPC, *h*ab, pAV, Car and Chl showed strong (>−0.9) but adverse relationships with the other variables. Furthermore, the variables of density, DPPH and FFA presented loads between −0.7 and −0.8, while OSI showed a load of less than 0.7. Figure 4B shows the locations of the different extraction methods that were used for sinami oil in this study.

The extraction methods were located along the four quadrants; therefore, the sinami oil that was obtained using EPE was characterized by a higher content of plant pigments (Car and Chl), while the oil that was extracted using CPE was represented by a higher antioxidant activity (DPPH and ABTS). Furthermore, the SFE sample showed higher values of L*, b*, C*ab and *h*ab and a lower pigment content compared to the EPE, CPE and UAE samples. The sinami oil that was obtained using UAE was characterized by higher levels of polyphenols. The EPE and CPE extraction methods had a similarity profile, according to the variables that were studied. The information that was provided by this PCA could contribute positively to the establishment of future studies that allow for the optimization of both EPE and CPE as part of the tech transfer in the sinami oil industrialization process.

## 4. Conclusions

In this study, an evaluation of the quality, total polyphenol content and antioxidant activity of sinami oil (*O. mapora* H. Karst) that was obtained using four extraction methods was studied. The quality parameters, chromatic parameters, plant pigments, total polyphenol content and antioxidant activity were all different for each extraction system. Significant differences were found for all of the variables, except the refractive index. Extraction using ultrasound presented a high peroxide value compared to that using CPE, EPE and SFE. The chemometric analysis that was illustrated by the PCA provided a visualization of the variables and the different extraction methods that were used to obtain the oil. In the PCA scores and load plots, the variables and extraction methods were distributed in all four quadrants. Consequently, the EPE and CPE extraction systems showed a certain similarity, with the former being characterized by a higher content of plant pigments and the latter presenting a higher antioxidant activity. However, further studies should be established to optimize sinami oil extraction using the cold press and expeller press methods in order to promote extraction on a larger scale and in turn allow for technology transfer to smaller producers. This paper comprises the first part of a larger study, which also includes the fatty acids, sterols, phenolic profiles, sensory characteristics, storage and characterization of by-products from the extraction of sinami oil using green extraction technologies.

## Figures and Tables

**Table 1:** Extraction yields and quality parameters of the sinami oils that were obtained using the four extraction systems.

Parameters	CPE	EPE	UAE	SFE
Extraction Yield (%)	5.52	6.99	15.64	7.45
Density (g/mL)	0.95 ± 0.01 ^a^	0.89 ± 0.02 ^b^	0.89 ± 0.01 ^b^	0.94 ± 0.01 ^ab^
Refractive Index (20 °C)	1.4690 ^a^	1.4691 ^a^	1.4650 ^b^	1.4690 ^a^
Specific Extinction (K232)	3.10 ± 0.05 ^b^	3.10 ± 0.08 ^b^	3.93 ± 0.13 ^a^	3.19 ± 0.11 ^b^
Specific Extinction (K270)	0.35 ± 0.01 ^c^	0.58 ± 0.03 ^b^	0.77 ± 0.03 ^a^	0.54 ± 0.02 ^b^
Extinction Coefficient Variation (∆K)	0.006 ^d^	0.018 ^b^	0.017 ^c^	0.018 ^a^
Free Acidity (%)	0.56 ± 0.09 ^b^	0.55 ± 0.09 ^b^	1.05 ± 0.08 ^a^	1.05 ± 0.08 ^a^
Peroxide Value (meq O_2_/kg)	22.76 ± 0.07 ^c^	22.66 ± 0.13 ^c^	43.58 ± 0.27 ^a^	25.88 ± 0.17 ^b^
pAV	4.06 ± 0.12 ^a^	4.13 ± 0.06 ^a^	3.96 ± 0.04 ^a^	2.11 ± 0.11 ^b^
TOTOX	53.65 ± 0.37 ^c^	53.58 ± 0.15 ^c^	95.08 ± 0.62 ^a^	55.97 ± 0.56 ^b^
Saponification Value (mg KOH/g)	202.17 ± 1.91 ^b^	202.75 ± 0.85 ^b^	196.08 ± 1.82 ^c^	219.75 ± 0.64 ^a^

The results are shown as the mean ± standard deviation. Different letters in the rows indicate significant differences, according to Duncan’s multiple range test at *p* < 0.05.

**Table 2:** Oxidative stability (OSI) of the sinami oils that were obtained using the four extraction systems and other oils from South American palm trees.

Oils	Extraction System	Stability Parameter			Reference
		T (°C)	Flow Rate (L/h)	OSI (h)	
Sinami	CPE	110	20	2.69 ± 0.17 ^b^	
Sinami	EPE	110	20	5.53 ± 0.46 ^a^	
Sinami	UAE	110	20	1.99 ± 0.32 ^b^	
Sinami	SFE	110	20	5.01 ± 0.08 ^a^	
Buriti (blend)	NS	110	9	18.30	[62]
Seje	CPE	120	20	4.28	[2]
Macaúba	NS	110	10	16.0	[60]
Bacaba	SFE	110	10	5.39	[4]
Patawa	CPE	100	10	2.79	[61]

Sinami (*Oenocarpus mapora*), buriti (*Mauritia flexuosa*), seje (*Jessenia bataua*), macaúba (*Acrocomia aculeata*), bacaba (*Oenocarpus bacaba*) and patawa (*Oenocarpus bataua*). Different letters in the columns indicate significant differences, according to Duncan’s multiple range test at *p* < 0.05. CPE, cold press extraction; EPE, expeller press extraction; UAE, ultrasound-assisted extraction; SFE, supercritical fluid extraction; NS, non-specified.

**Table 3:** Chromatic properties of the sinami oils that were obtained using the four extraction systems.

Extraction System	Input Color Value	L*	a*	b*	C*ab	*h*ab	View
CPE	rgb 139 149 3	58.96	−18.73	61.88	64.65	73.16	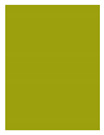
	rgb 146 148 7	59.43	−15.21	61.78	63.63	76.16	
	rgb 144 149 7	59.48	−16.76	61.84	64.07	74.84	
	rgb 158 162 14	64.23	−16.90	64.83	66.99	75.39	
	rgb 161 165 15	65.34	−17.27	65.60	67.83	75.25	
	rgb 160 164 18	64.90	−17.07	64.77	66.98	75.26	
	rgb 165 169 16	66.95	−17.54	66.99	69.25	75.32	
	rgb 162 167 15	66.04	−17.62	66.23	68.53	75.10	
	rgb 171 173 17	68.42	−16.63	68.26	70.25	76.30	
	rgb 140 149 3	59.02	−18.29	61.91	64.56	73.55	
EPE	rgb 137 143 4	56.97	−16.37	60.06	62.26	74.75	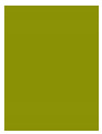
	rgb 151 158 7	62.54	−17.99	64.37	66.83	74.38	
	rgb 139 145 4	57.78	−16.77	60.74	63.02	74.57	
	rgb 138 141 6	56.45	−15.24	59.42	61.35	75.61	
	rgb 133 137 4	54.90	−15.21	58.44	60.39	75.41	
	rgb 153 158 7	62.65	−17.25	64.51	66.78	75.03	
	rgb 143 150 2	59.43	−17.58	62.48	64.90	74.29	
	rgb 130 137 2	54.61	−16.41	58.35	60.61	74.29	
	rgb 131 139 1	55.41	−17.46	59.21	61.73	73.57	
	rgb 132 140 1	55.78	−17.71	59.49	62.07	73.42	
UAE	rgb 155 167 51	65.51	−19.06	55.37	58.56	71.00	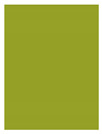
	rgb 144 154 41	60.87	−18.15	54.56	57.50	71.60	
	rgb 146 155 38	61.52	−18.12	56.27	59.11	72.15	
	rgb 152 165 38	64.67	−20.17	59.13	62.48	71.17	
	rgb 147 159 33	62.50	−19.38	58.78	61.89	71.75	
	rgb 148 161 31	63.39	−20.38	59.96	63.32	71.23	
	rgb 149 161 31	63.20	−19.89	59.97	63.18	71.65	
	rgb 152 164 38	64.39	−19.96	59.08	62.36	71.33	
	rgb 154 165 48	65.04	−19.26	56.20	59.41	71.09	
	rgb 144 154 42	60.90	−17.98	54.04	56.95	71.60	
SFE	rgb 168 174 135	83.48	−10.23	73.96	74.67	82.12	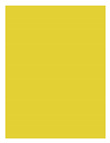
	rgb 163 170 129	82.12	−9.99	72.38	73.05	82.19	
	rgb 156 164 122	83.05	−10.01	73.45	74.12	82.24	
	rgb 171 177 131	82.31	−10.04	72.36	73.05	82.10	
	rgb 167 174 128	83.64	−10.19	74.66	75.36	82.23	
	rgb 165 173 124	82.15	−10.11	72.38	73.08	82.05	
	rgb 175 180 131	83.10	−10.09	73.59	74.28	82.19	
	rgb 166 173 122	81.97	−10.09	72.57	73.17	82.09	
	rgb 169 175 124	82.14	−9.81	72.13	72.80	82.26	
	rgb 161 168 116	83.06	−10.10	74.16	74.85	82.24	
CPE		63.28 ± 3.67 ^b^	−17.20 ± 0.97 ^b^	64.41 ± 2.42 ^b^	66.67 ± 2.34 ^b^	75.0.3 ± 1.00 ^b^	
EPE		57.65 ± 2.97 ^c^	−16.80 ± 0.99 ^b^	60.71 ± 2.30 ^c^	62.99 ± 2.38 ^c^	74.53 ± 0.71 ^b^	
UAE		63.20 ± 1.71 ^b^	−19.23 ± 0.89 ^a^	57.34 ± 2.28 ^d^	60.48 ± 2.42 ^d^	71.46 ± 0.36 ^c^	
SFE		82.70 ± 0.63 ^a^	−10.06 ± 0.12 ^c^	73.16 ± 0.91 ^a^	73.05 ± 0.91 ^a^	82.17 ± 0.07 ^a^	

Different letters in the columns indicate significant differences, according to Duncan’s multiple range test at *p* < 0.05.

**Table 4:** Pigments, polyphenols and antioxidant activity of the sinami oils that were obtained using the four extraction systems.

Extraction System	Plant Pigments		Total Phenolics	DPPH (IC_50_), mg/mL	ABTS (IC_50_), mg/mL
	Carotenoids	Chlorophylls			
CPE	47.13 ± 0.02 ^b^	56.24 ± 0.10 ^b^	89.03 ± 5.87 ^d^	5.70 ± 0.32 ^c^	13.12 ± 0.10 ^c^
EPE	51.62 ± 0.65 ^a^	109.56 ± 1.14 ^a^	116.72 ± 1.96 ^c^	3.77 ± 0.37 ^ab^	11.86 ± 0.02 ^b^
UAE	42.32 ± 0.04 ^c^	111.67 ± 0.68 ^a^	615.18 ± 3.92 ^a^	3.24 ± 0.33 ^a^	7.81 ± 0.43 ^a^
SFE	12.29 ± 0.29 ^d^	3.48 ± 1.25 ^c^	200.49 ± 4.90 ^b^	4.58 ± 0.21 ^b^	11.28 ± 0.11 ^b^

Different letters in the columns indicate significant differences, according to Duncan’s multiple range test at *p* < 0.05.

## Data Availability

All data are contained within the manuscript.

## Supplementary Materials

The following supporting information can be downloaded at: https://www.mdpi.com/article/10.3390/foods11101518/s1, Figure S1: Static extraction of sinami oil.
