# Supplementary material for: Comparison of Four Oil Extraction Methods for Sinami Fruit (Oenocarpus mapora H. Karst): Evaluating Quality, Polyphenol Content and Antioxidant Activity
Source: Foods. 2022 May 23;11(10):1518. doi: 10.3390/foods11101518 (PMC9141738; doi:10.3390/foods11101518)

foods

MDPI

Article

Comparison of Four Oil Extraction Methods for Sinami Fruit (*Oenocarpus mapora* H. Karst): Evaluating Quality, Polyphenol Content and Antioxidant Activity

Ana María Muñoz [1,2], Sandra Casimiro-Gonzales [1], Raquel B. Gómez-Coca [3], Wenceslao Moreda [3], Ivan Best [1,2], María Isabel Cajo-Pinche [4], Juan Francisco Loja [5], Elena Ibañez [6], Alejandro Cifuentes [6] and Fernando Ramos-Escudero [2,7,*]

1 Instituto de Ciencias de Los Alimentos y Nutrición, Universidad San Ignacio de Loyola (ICAN-USIL), Campus Pachacamac, Sección B, Parcela 1, Fundo La Carolina, Pachacamac, Lima 15823, Peru; amunoz@usil.edu.pe (A.M.M.); scasimiro@usil.edu.pe (S.C.-G.); ibest@usil.edu.pe (I.B.)
2 Unidad de Investigación en Nutrición, Salud, Alimentos Funcionales y Nutraceúticos, Universidad San Ignacio de Loyola (UNUSAN-USIL), Av. La Fontana 750, Lima 15024, Peru
3 Instituto de la Grasa, CSIC, Campus Universidad Pablo de Olavide, Building 46, Ctra. de Utrera km 1, 41013 Sevilla, Spain; raquel.coca@ig.csic.es (R.B.G.-C.); wmoreda@ig.csic.es (W.M.)
4 Carrera Profesional de Ingeniería Agroindustrial, Universidad Nacional Amazónica de Madre de Dios (UNAMAD), Jr. Jorge Chávez 1160, Puerto Maldonado 17001, Peru; cajo_maisa@hotmail.com
5 Asociación para la Conservación de la Cuenca Amazónica (ACCA), Madre de Dios 17001, Peru; jloja@conservacionamazonica.org
6 Foodomics Laboratory, Instituto de Investigación en Ciencias de la Alimentación (CIAL, CSIC-UAM), Nicolás Cabrera 9, Campus de Cantoblanco, 28049 Madrid, Spain; elena.ibanez@csic.es (E.I.); a.cifuentes@csic.es (A.C.)
7 Facultad de Ciencias de la Salud, Universidad San Ignacio de Loyola, Av. La Fontana 750, Lima 15024, Peru
* Correspondence: dramos@usil.edu.pe or diomedes.fernando@gmail.com

Figure S1. Static extraction of sinami oil

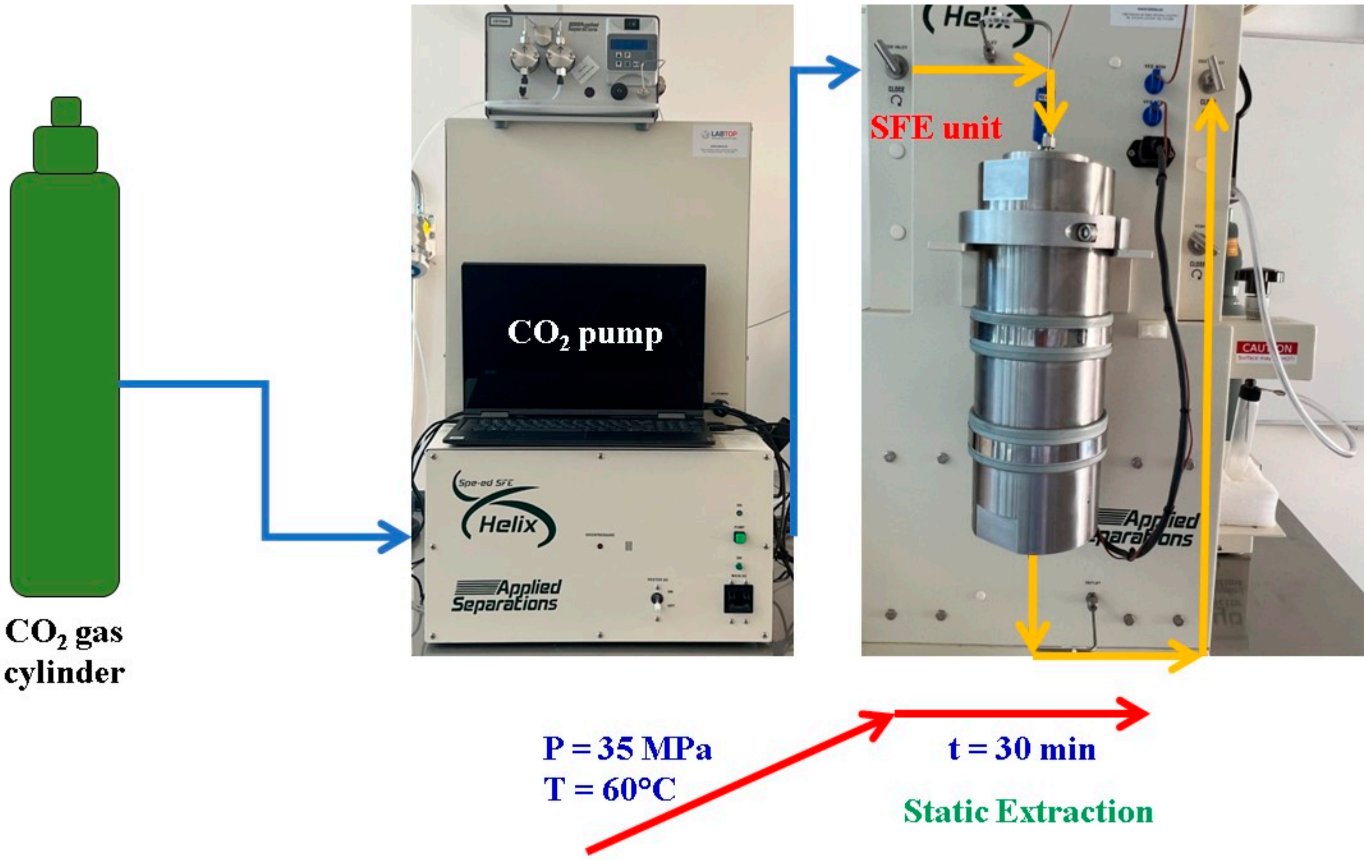

Supplement: Supplementary file 1 [file foods-11-01518-s001.zip › foods-1714045-supplementary.pdf]
